# Supplementary material for: Greenhouse Gas (GHG) Emissions from Honey Production: Two-Year Survey in Italian Beekeeping Farms
Source: Animals (Basel). 2023 Feb 20;13(4):766. doi: 10.3390/ani13040766 (PMC9952000; doi:10.3390/ani13040766)
Supplement: Supplementary file 1 [file animals-13-00766-s001.zip › animals-2104482-supplementary.pdf]

animals

animals-2104482 Supplementary Materials

Table S1. Life Cycle Inventory (CI) inputs and outputs.

Life Cycle Phases	Inputs	Outputs
Hive placement (winter)	Beehives<br>Wooden frame<br>Beehive paint<br>Supplemental feeding<br>Beeswax<br>Medication<br>Electricity<br>Beekeeping equipment<br>Transportation	Beehives with honeybees
Hive placement (summer)	Beehives<br>Wooden frame<br>Beehive paint<br>Supplemental feeding<br>Beeswax<br>Medication<br>Beekeeping equipment<br>Transportation	Beehives filled with honey<br>Co-products:<br>honeycombs
Honey extraction	Uniform<br>Gloves<br>Lab coats<br>Electricity<br>Water<br>Machines<br>Filters	Honey<br>Co-products:<br>beeswax,<br>propolis,<br>royal jelly

Table S2. Life Cycle Inventory of Hive Placement (winter and summer) phase.

		2020						2021					
**Inputs**	**Unit**	**Farm 1**	**Farm 2**	**Farm 3**	**Farm 4**	**Farm 5**	**Farm 6**	**Farm 1**	**Farm 2**	**Farm 3**	**Farm 4**	**Farm 5**	**Farm 6**
Transport for hive placement (winter)	km/hive	109.0	22.0	12.0	0	0	0	110.0	5.2	16.0	0	0	0
Transport for hive placement (summer)	km/hive	41.0	23.0	14.0	0	0	0	12.0	5.2	14.0	0	0	0
Medication	gr/hive	66.0	66.0	51.0	2.4	53.0	28.7	356.1	53.2	1002.6	1.1	81.8	28.3
Supplemental feeding	kg/hive	15.0	13.0	0.7	1.2	2.0	1.5	15.0	17.0	4.0	10	3	1.5
Electricity *(Maintain the frames)*	kWh/hive	3.54	3.52	4.03	1.67	3.55	3.54	0.94	0.24	3.06	0.1	0.9	1.1
Beekeeping equipment	kg/hive	1.2	1.2	0.4	0.1	0.4	0.1	0.7	1.6	0.9	0,.6	0.8	0.1
Wood (*Wooden frame*)	m^3/hive	0.1	0.1	0.1	0.1	0.1	0.1	0.1	0.1	0.1	0.1	0.1	0.1
Beehive Paint	kg/hive	0.1	0.1	0.1	0.1	0.1	0.1	0.1	0.1	0.1	0.1	0.1	0.1
**Outputs**	**Amount**	**Unit**											
Hive	*One*	*Item*											

Table S3. Life Cycle Inventory of Honey extraction phase.

		2020						2021					
**Inputs**	**Unit**	**Farm 1**	**Farm 2**	**Farm 3**	**Farm 4**	**Farm 5**	**Farm 6**	**Farm 1**	**Farm 2**	**Farm 3**	**Farm 4**	**Farm 5**	**Farm 6**
Water	l/honey	30.0	29.0	61.0	4.4	22.4	17.4	14.5	25.0	66.0	6.4	14.6	0.3
Electricity *(Honey extraction)*	kWh/honey	0.7	0.5	0.6	0.3	0.5	0.4	0.4	0.8	1.2	0.7	0.5	0.6
Beekeeping Clothing *(Uniform, gloves, lab coat)*	gr/honey	0.1	0.1	0.1	0.1	0.1	0.1	0.1	0.1	0.1	0.1	0.1	0.1
Honey Extraction equipment (*Machines*)	kg/honey	0.1	0.2	0.2	0.2	0.2	0.1	0.2	0.5	0.4	0.2	0.6	1.1
**Outputs**	**Amount**	**Unit**											
Honey	*One*	*kilogram*											

Table S4. Collecting meteorological data.

	Temperature	Temperature	Precipitation	Precipitation	Days with Rain	Days with Rain
	Mean	*Mean*	*Millimeters*	*Millimeters*	*Number*	*Number*
	**2020**	**2021**	**2020**	**2021**	**2020**	**2021**
44°25′ N 10°12′ E						
Jan	10.1	4.3	63.8	261.0	8	13
Feb	12.2	7.9	65.2	134.4	8	11
Mar	12.1	8.6	133.8	26.6	9	5
Apr	17.7	10.2	49.0	129.2	2	10
May	21.7	13.2	85.0	160.8	7	15
Jun	23.1	20.4	183.2	26.8	8	4
Jul	27.5	22.3	23.2	40.6	3	5
Aug	27.6	22.1	151.0	90.2	6	8
Sep	23.8	19.7	58.2	275.6	8	6
Oct	16.0	13.4	241.2	93.8	13	6
Nov	14.1	9.7	43.4	154.0	8	12
Dec	8.7	6.3	424.6	139.4	21	12
	17.9	13.2	1521.6	1532.4	101	107
44°40′ N 10°27′ E						
Jan	4.4	1.7	13.0	207.6	5	14
Feb	7.5	5.0	14.8	82.4	4	9
Mar	5.6	6.0	155.8	6.6	15	3
Apr	10.4	8.0	65.6	110.4	5	12
May	14.8	13.2	98.2	77.8	10	10
Jun	17.5	20.0	96.6	56.6	13	3
Jul	20.7	21.7	52.4	17.8	6	6
Aug	21.5	21.0	177.4	42.0	7	4
Sep	16.6	17.3	69.0	168.2	10	5
Oct	10.5	10.2	172.4	57.2	17	9
Nov	7.3	6.5	32.0	181.8	5	19
Dec	3.2	3.7	378.6	79.4	21	14
	11.7	11.2	1325.8	1087.8	118	108
43°78′ N 11°32′ E						
Jan	7.9	6.4	51.8	144.2	5	15
Feb	10.1	9.7	32.0	79.2	5	7
Mar	10.4	10.5	57.6	4.0	5	1
Apr	14.4	12.3	36.6	83.2	4	7
May	18.9	16.7	61.6	57.0	7	11
Jun	21.4	23.6	41.8	12.4	8	3
Jul	25.2	25.6	24.6	14.6	2	1
Aug	26.3	25.7	26.8	17.6	3	3
Sep	22.1	21.8	38.0	68.8	8	6
Oct	14.6	15.6	169.4	33.4	10	3
Nov	12.3	12.3	25.4	111.4	5	14
Dec	8.2	7.7	160.0	167.2	17	4
	16.0	15.7	725.6	793.0	79	75
44°66′ N 11°33′ E						
Jan	3.0	2.0	21.6	32.8	16	17

Feb	6.8	6.4	1.2	16.0	6	9
Mar	8.5	7.5	18.8	9.0	11	5
Apr	12.8	10.6	23.2	46.6	3	1
May	18.2	16.2	15.2	45.0	8	3
Jun	20.8	23.0	70.4	27.4	10	4
Jul	23.8	25.0	62.8	51.8	9	6
Aug	24.8	24.6	128.4	62.8	10	6
Sep	20.2	20.5	66.8	24.8	13	6
Oct	13.3	13.2	65.2	11.2	17	6
Nov	8.6	9.1	24.2	88.0	14	20
Dec	5.0	2.4	151.6	25.2	21	16
	13.8	13.4	649.4	440.6	138	99
44°81′ N 10°80′ E						
Jan	3.4	1.7	20.4	50.2	17	17
Feb	7.6	6.3	1.2	23.8	5	9
Mar	8.9	7.8	38.2	1.0	8	5
Apr	14.0	11.0	22.6	59.4	5	14
May	19.8	16.6	25.0	56.2	9	8
Jun	21.7	23.3	27.8	48.4	8	4
Jul	24.4	24.6	66.2	15.8	6	6
Aug	25.2	23.7	77.2	29.6	7	6
Sep	21.3	19.7	66.0	42.2	10	6
Oct	13.3	12.3	90.8	28.0	13	6
Nov	8.8	8.6	20.0	108.2	11	19
Dec	4.5	1.9	167.6	65.0	11	15
	14.4	13.1	623.0	527.8	110	115
44°50′ N 10°45′ E						
Jan	2.7	1.7	24.4	50.2	18	17
Feb	7.1	6.3	1.0	23.8	7	8
Mar	8.4	7.8	32.4	1.0	12	3
Apr	12.9	11.0	39.4	59.4	6	8
May	18.4	16.6	22.6	56.2	6	8
Jun	20.9	23.3	80.8	48.4	9	2
Jul	23.9	24.6	86.4	15.8	9	6
Aug	24.6	23.7	141.2	29.6	6	5
Sep	19.7	19.7	36.2	42.2	9	8
Oct	12.8	12.3	87.6	28.0	10	6
Nov	8.3	8.6	21.2	108.2	18	23
Dec	4.6	1.9	196.2	65.0	14	21
	13.7	13.1	769.4	527.8	124	115

Table S5. Contributions to Carbon Footprint (% of total impact) of honey production and yield in the two years of survey.

	**2020**						**2021**					
	**Hive Management (Spring-Summer Season)**		**Hive Management (Winter Season)**		**Honey Extraction**		**Hive Management (Spring-Summer Season)**		**Hive Management (Winter Season)**		**Honey Extraction**	
	**Kg CO_2 e/kg of Honey**	**% Total Impact**	**Kg CO_2 e/kg of Honey**	**% Total Impact**	**Kg CO_2 e/kg of Honey**	**% Total Impact**	**Kg CO_2 e/kg of Honey**	**% Total Impact**	**Kg CO_2 e/kg of Honey**	**% Total Impact**	**Kg CO_2 e/kg of Honey**	**% Total Impact**
Farm 1	1.19	55%	0.59	27%	0.39	18%	1.72	41%	2.23	53%	0.24	6%
Farm 2	0.87	48%	0.56	32%	0.35	20%	1.22	42%	1.22	42%	0.47	16%
Farm 3	0.68	43%	0.44	28%	0.45	28%	0.9	42%	0.46	21%	0.81	37%
Farm 4	0.026	6%	0.14	33%	0.25	61%	0.25	26%	0.26	26%	0.46	48%
Farm 5	0.007	2%	0.17	37%	0.28	61%	0.03	4%	0.36	47%	0.38	49%
Farm 6	0.006	2%	0.12	34%	0.24	64%	0.004	2%	0.1	19%	0.41	79%
